# 

**Published:** 1894-02-10

**Authors:** 


### CONTENTS.

**Should Civilization Suppress Quackery?** - - - - 319
**Around the Hospitals** - - - - - - - - - - 320
**On Modern Progress in Ophthalmic Medicine and Surgery.** By ROBERT BRUDENELL CARTER, F.R.C.S. - 321
**Art and Disease.** V.—The Lame and the Blind - - - - 322
**Medical Progress and Hospital Clinics**—Inflamed Eyes - 327
Modern Methods of Treatment of Compound Fracture - - 328
MEDICAL PROGRESS—Diseases of the Lung - - - - - 329
PROGRESS IN SURGERY - - - - - - - - - - 331
NEW APPLIANCES AND THINGS MEDICAL—Kingzett's Improved Sulphur Fumigating Candles - - - - - - - - 333
NEW DRUGS AND PREPARATIONS.—Trional and Tetronal—Professor Unna's Gelatines - - - - - - - - 334
**Modern Aspects of Pernicious Anæmia** - - - - 323
**The Field of Science** - - - - - - - - - 324
**Annotations.**—Toothbrush Drill—The International Sanitary Conference—Shall London Have a University? - - - 325
**Notes and News** - - - - - - - - - - - 326
**The Institutional Workshop—**
HOSPITAL ADMINISTRATION—New Hospitals v. Hospital Extension for London - - - - - - - - - - 335
THE STORY OF THE INSANE FROM YEAR TO YEAR - - - 336
PRACTICAL DEPARTMENTS—Ward Fittings at Middlesex Hospital 337
HOSPITAL FESTIVALS AND MEETINGS - - - - - - 337
CONSTRUCTION NOTES - - - - - - - - - 337
**Scraps and Gleanings** - - - - - - - - - 338

### EXTRA SUPPLEMENT.—THE NURSING MIRROR.

**News from the Nursing World.**—A Royal Visit—The Royal British Nurses' Association—Chelsea Hospital for Women—No New Thing—Young Workers Wanted—More Cheap Poisons—Private Nursing As It Is—Two Coventry Institutions—A Lucky Hit—Queen's Nurses in the Isle of Wight — Comfortable Isolation —Very Hard Boards—Hôpital Boucicaut—Invalids Abroad—Short Items - - clxxxi
**On Nursing the Nervous and Insane.** V.—Hypnotism clxxxiii
**Nursing in India—Minor Appointments** - - - clxxxiii
**District Nursing.**—VI. District Hygiene - - - - clxxxiv
**The Nurses' Co-operation** - - - - - - - - clxxxiv
**The Nursing of Ovariotomy Patients.** II.—The Nourishment - - - - - - - - - - - clxxxv
**Nursing in the United States—Presentations** - clxxxvi
**Everybody's Opinion.** — Nursing Small-pox — Nursing Ovariotomy Patients—Midwives' Association - - - clxxxvii
**Novelties and New Preparations** - - - - - clxxxvii
**Women's Work.**—The Resident Governess - - - - clxxxviii
**Muses' Looking Glass.**—Women Writers and Their Ways clxxxix
**The Book World for Women and Nurses** - - - cxc

